# In the Right Place, at the Right Time: Spatiotemporal Conditions Determining Plasma Cell Survival and Function

**DOI:** 10.3389/fimmu.2019.00788

**Published:** 2019-04-24

**Authors:** Randall L. Lindquist, Raluca A. Niesner, Anja E. Hauser

**Affiliations:** ^1^Immunodynamics, Deutsches Rheuma-Forschungszentrum Berlin, A Leibniz Institute, Berlin, Germany; ^2^Biophysical Analysis, Deutsches Rheuma-Forschungszentrum Berlin, A Leibniz Institute, Berlin, Germany; ^3^Fachbereich Veterinärmedizin, Institute of Veterinary Physiology, Freie Universität Berlin, Berlin, Germany; ^4^Department of Rheumatology and Clinical Immunology, Charité-Universitätsmedizin Berlin, Berlin, Germany

**Keywords:** plasma cells, survival, bone marrow, inflammation, intravital 2P microscopy, tissue niches, metabolism

## Abstract

Plasma cells (PCs), the B lineage cells responsible for producing and secreting antibodies (Abs), are critical cellular components of the humoral immune system. While most of the antibody-secreting cells in the body have a rather short lifetime of a few days, some of them can become long-lived and persist in the body over the entire life span of an individual. The majority of these long-lived plasma cells secretes protective antibodies against pathogens, and are thereby crucial for the humoral component of immunological memory. The generation of these protective antibody-secreting cells can be triggered by an exposure to pathogens, and also by vaccination. Although the majority of plasma cells are protective, sometimes long-lived plasma cells produce autoreactive antibodies, which contribute to the pathogenesis and perpetuation of chronic autoimmune diseases, including lupus erythematosus, rheumatoid arthritis, or multiple sclerosis. In order to promote the formation of protective antibody-secreting cells and to target pathogenic plasma cells, it is crucial to understand the signals which promote their longevity and allow them to exert their function. In recent years, it has become clear that plasma cells depend on extrinsic factors for their survival, leading to the concept that certain tissue microenvironments promote plasma cell retention and longevity. However, these niches are not static structures, but also have dynamic features with respect to their cellular composition. Here, we review what is known about the molecular and cellular composition of the niches, and discuss the impact of dynamic changes within these microenvironments on plasma cell function. As plasma cell metabolism is tightly linked to their function, we present new tools, which will allow us to analyze metabolic parameters in the plasma cell niches *in vivo* over time.

## Antibody Secreting Cells—the Cellular Basis of Humoral Immunity

Humoral immunity is mediated by antibodies, produced by cells highly specialized to synthesize and secrete large quantities of proteins (1). Antibody-secreting cells are traditionally divided into plasmablasts and plasma cells, on the basis of their capacity to further proliferate; as the name suggests, plasmablasts retain the capacity to proliferate, while plasma cells are considered to be post-mitotic. While most of the antibody-secreting cells in the body have a rather short lifetime of a few days, some of them can become long-lived and persist in the body over the entire life span of an individual, as described 20 years ago (2, 3), thereby providing the cellular basis of long-term antibody responses.

## Plasma Cell Generation, Migration, and Survival Under Protective/Physiologic Conditions

Plasma cell differentiation from activated B cells begins in secondary lymphoid organs. During the early phases of an immune response, after the initial B cell activation has taken place but before the formation of germinal centers, a first wave of antigen-specific plasma blasts is generated. These cells are generally considered short-lived (4) and relocate apart from the B cell zones, in the splenic red pulp (RP) (5), or the medullary cords (MC) of lymph nodes (6), respectively. These areas contain Gr1^int^CD11b^hi^F4/80^+^ macrophages which are a source of APRIL, a potent survival factor for plasma cells. Under conditions where no germinal centers are formed, e.g., in immune responses to T cell-independent antigens or when germinal center formation is experimentally blocked, B cells differentiate into extrafollicular plasma blasts, which later can become long-lived plasma cells (7), in agreement with the concept that extrinsic factors determine the capacity of plasma cells to survive (5).

On their way from the B cell zone to the medullary cords, migratory plasma blasts pass by a microenvironment rich in the growth factor IL-6, stemming from perivascular CD11c+ cells in the T cell zone. Directed migration from the B cell follicles to the medullary cords (8) is guided by a gradient of CXCL12, produced by medullary fibroblastic reticular cells (9). These specialized stromal cells are also a local source of plasma cell survival factors IL-6, BAFF, and APRIL, and synergize with the myeloid cells in promoting plasma cell survival at this location. Upon settling in the medullary cords, they become mature plasma cells, and cease their migration (10) and proliferation (6).

During later phases of primary as well as recall T-dependent immune responses, plasma blasts are generated during germinal center reactions. Germinal centers develop in B cell follicles in the course of T-dependent responses, and represent unique microanatomical structures, where B cells proliferate and encounter antigen deposited on the surface of follicular dendritic cells in the germinal center light zone (11). After B cell receptor-mediated antigen uptake, they present the antigen to T cells, and in turn receive T cell help. Successful antigen presentation to T cells induces further B cell proliferation and somatic hypermutation of their immunoglobulin (Ig) variable genes in the dark zone, followed by the selection of B cells with higher affinity B cell receptors (12). Multiple rounds of B cell proliferation and selection result in the affinity maturation of the B cell immune response, and in the output of memory B cells and antibody secreting cells. Plasma blast differentiation is initiated by antigenic stimulation in the light zone, and their further maturation occurs after they have localized to the dark zone (13). A recent study has identified the germinal center-T zone interface (GTI) as an important site for plasma cell maturation during the early maturation phases of the GC response, using a model of primary immunization (14). The GTI shares characteristics with the RP/MC microenvironment as it is rich in IL-6, APRIL and CXCL12, produced by stromal cells in this region. In addition, IL-21 from Tfh cells, known to promote plasma blast differentiation, is abundant in these areas. Plasma blasts leave the GTI migrating in the direction of the medullary cords/red pulp areas (14). Whether these migrating cells feed directly into the short-lived pool or leave the SLOs to become long-lived in the bone marrow is currently unclear. Using pulse-chase experiments, Weisel et al. (15) found that the majority of long-term resident plasma cells in the bone marrow were actually derived from late GC phases. However, antigen-specific plasma cells accumulate as early as day 3 in the bone marrow (16, 17). This may be explained by the possibility that plasma cells of a higher affinity can gradually replace the earlier generated low-affinity ones in the bone marrow, but this remains to be formally proven. Further, the mechanism by which plasma cells could compete on the basis of affinity remains unclear, as plasma cells lack membrane expression of their antigen receptor, and antigen is not retained in the bone marrow to a similar extent as on follicular dendritic cells. Nevertheless, a bulk/first wave of plasma blasts leaves the SLO and migrates to the bone marrow in the time frame of a week after immunization in mice (16, 17), and a peak of antigen-specific plasma blasts can be detected in the blood of humans at day 6 and 7 after immunization (18), indicating a similar time course in humans.

There are distinct molecular requirements for cellular entry or exit into tissue: the receptor for sphingosine-1-phosphate S1PR1 (19), whose expression on plasma blasts is modulated by mir-17-92 (20), and integrin receptor beta 2 (21) are required on plasma blasts to leave lymph nodes, while the chemokine receptor CXCR4 (16, 17) is required for bone marrow homing. Plasma blasts also transiently respond to CXCR3-ligands, however, at what stage of plasma blast migration these chemokines play a role is not known (17).

The CXCR4 ligand CXCL12 is produced in high amounts by bone marrow stromal cells (22). Notably, plasma blasts deficient in the transcription factor c-Myb exhibit a deficiency in migration toward CXCL12 gradients *in vitro* and mislocalize to the T cell zone in the spleen, indicating that they are not able to reach the red pulp (23). Thus, CXCR4 seems to not only control access to “exit points” for extravasation from secondary lymphoid organs, but migration to specific domains within lymphoid tissues. The nature of these egress sites has not yet been defined in detail. Plasma blasts in the red pulp occur in clusters, which indicates that these sites are present within the sinusoidal vessel structures of this compartment. Shp1 deficient plasma blasts are able to migrate to the red pulp, but do not form clusters and are impaired in their bone marrow homing capability due to an enhanced binding to integrin α4β1 to its ligand VCAM-1, which results in an impaired capacity to migrate (24). Integrin α4β1 (VLA-4) has been implied in multiple aspects of plasma cell biology, and seemingly contradictory results may be explained by its different functions in varying microenvironments. For example, integrin β1 activation by the cochaperone Mzb1 has been shown to contribute to the relocation of plasma blasts (25), however, this seems to mainly affect their entry into the bone marrow, not their egress from SLOs. CXCL12 has also been shown to activate α4β1 (26), and VCAM-1 mediated stimulation of α4β1 impacts on the survival of plasma cells (27). This particular function seems to depend on CD37, which regulates the membrane distribution of α4β1, thereby enabling signaling via the Akt survival pathway (28).

## Microenvironments of Plasma Cell Niches in the Bone Marrow

It has long been known that plasma cells accumulate in the bone marrow (29). Long-lived plasma cells were first described in this organ (2, 3), and as it is the primary locus of humoral memory, the bone marrow microenvironment has been the most intensively studied plasma cell niche.

The entry points and routes which plasma cells use to enter the bone marrow from the blood are not completely identified yet, but they are likely similar to the ones used by hematopoietic stem and progenitor cells (HSPCs). Bone marrow vasculature comprises small arterioles, which regulate the blood flow into the parenchyma. These vessels progressively increase their diameter and connect to a network of sinusoids, which are characterized by large lumina (30, 31). The fenestrated endothelia and the discontinuous structure of their underlying basement membrane (32), in combination with low blood flow velocities make this vascular compartment the preferred entry site for cells, as has been shown for HSPCs (33).

Plasma cell survival crucially depends on a combination of extrinsic signals, among them adhesion molecules (27). After crossing the endothelium, plasma blasts migrate to specialized microenvironments (“niches”) in the bone marrow parenchyma. Their migration is directed by stromal-derived factor 1α (CXCL12). Upon arrival at its niche, a motile plasma blast loses its responsiveness to chemokines (17) and docks onto stromal cells (34, 35). The newly arrived plasma blasts then becomes sessile, and remains constantly in close contact with the stromal cell (36). This contact seems to be based on α4β1 (VLA-4) and αLβ2 (LFA-1) on plasma cells interacting with their respective ligands on stromal cells, as only the combined blockade of both adhesion molecules by antibodies has been shown to effectively deplete plasma cells from the bone marrow (37). The stromal cells on which plasma cells colocalize have been shown to be VCAM-1^+^ (34), however, a recent study provided evidence that fibronectin, another ligand of α4β1 integrin, also mediates plasma cell survival (38). Less is known about which of the ligands for αLβ2 (of which there are 6: ICAM1-5 and JAM-A) are of relevance for plasma cells in their niches, and fibronectin also binds CD44, which has been shown to mediate plasma cell survival *in vitro* (27). It is currently not known whether the stromal cells, which support plasma cell niches constitute a functionally specialized subset within the bone marrow stroma. Although most reticular bone marrow stromal cells express CXCL12, there is evidence for considerable heterogeneity among the stromal compartment (39), and it will be important to analyze the relationship of hematopoietic cell types to these various stromal subsets *in situ* using multiplexed microscopy (40). The term “stromal cell” is known to encompass a wide range of cell types—in the lymph node, for example, single-cell RNA sequencing was used to define 9 different stromal cell populations (41). Four of these were previously known (follicular dendritic cells, marginal reticular cells, perivascular cells, and T-zone reticular cells), while the rest had not been previously appreciated or defined. Given the wide range of cell types and processes that occur within the bone marrow, it is unlikely that bone marrow stromal cells will be less heterogenous than lymph node stromal cells.

The niches provide plasma cells not only with signals for their adhesion, but also directly promote PC survival. BCMA on plasma cells is a crucial mediator of survival signals (42) via its ligands APRIL and BAFF (43). BCMA signaling regulates the expression of the anti-apoptotic protein Mcl-1 in bone marrow plasma cells (44). Multiple different cellular sources of APRIL have been reported in the bone marrow. Eosinophils have been shown to produce APRIL and also IL-6, another survival factor for plasma cells, and support plasma cell survival *in vitro* (45). The same group also showed that bone marrow plasma cell numbers are reduced in eosinophil-deficient or –depleted mice to about half the numbers present in controls. In contrast, two recent studies by other labs (46, 47) found that eosinophils were actually dispensable for plasma cell survival in the bone marrow. These discrepancies could be in part explained by environmental factors, for example differences in the microbiome of animals, as the two latter studies used littermate controls, which Chu et al. did not. In addition, these findings indicate that more than one cell type may contribute to BCMA-dependent plasma cell survival. Indeed, APRIL and IL-6 have also been shown to be produced by megakaryocytes in the bone marrow (48). Plasma cell numbers are reduced in megakaryocyte-deficient c-mpl mice, whereas raising megakaryocyte numbers pharmacologically is accompanied by an increase in plasma cell numbers, suggesting that megakaryocyte-derived APRIL and IL-6 are physiologically relevant to bone marrow plasma cells.

Although most studies have focused on APRIL, the other ligand of BCMA, BAFF, likely also plays a role in plasma cell survival, as a reduction in bone marrow plasma cells is only observed in APRIL^−/−^ mice, if BAFF is blocked as well (43). The redundancy of the hematopoietic cell types contributing to plasma cell niches is further underlined by reports of several other cell types, which have been reported to support their survival via different mechanisms: Ly6C^hi^ monocytes have been shown to produce APRIL and co-localize with bone marrow plasma cells in mice (49), and myeloid precursors are a major source of APRIL in humans (50). In mice, basophils have been shown to support plasma cell survival by producing IL-4 and IL-6 *in vitro* and *in vivo* (51). Dendritic cells have also been shown to make functional contacts with bone marrow plasma cells: CD80/86 on the DC surface interacts with CD28 on bone marrow plasma cells, which promotes their survival by activation of the Vav and NF-κB pathways (52). Consequently, CD28^−/−^ mice have a reduction in bone marrow plasma cells (53). By intravital microscopy, bone marrow plasma cells have been shown to form contacts with CD11c^+^ cells as well as T regulatory cells (54), but the mechanisms by which these two cell types regulate niches are not fully elucidated. Deletion of CTLA-4 in the Tregs resulted in an increase in plasma cell numbers, which may indicate that they are able to modulate the function of the niche dendritic cells. On the other hand, depletion of Tregs induced by systemic infection caused a reduction of the bone marrow plasma cell population, indicative of their role in plasma cell maintenance.

**Figure 1:**
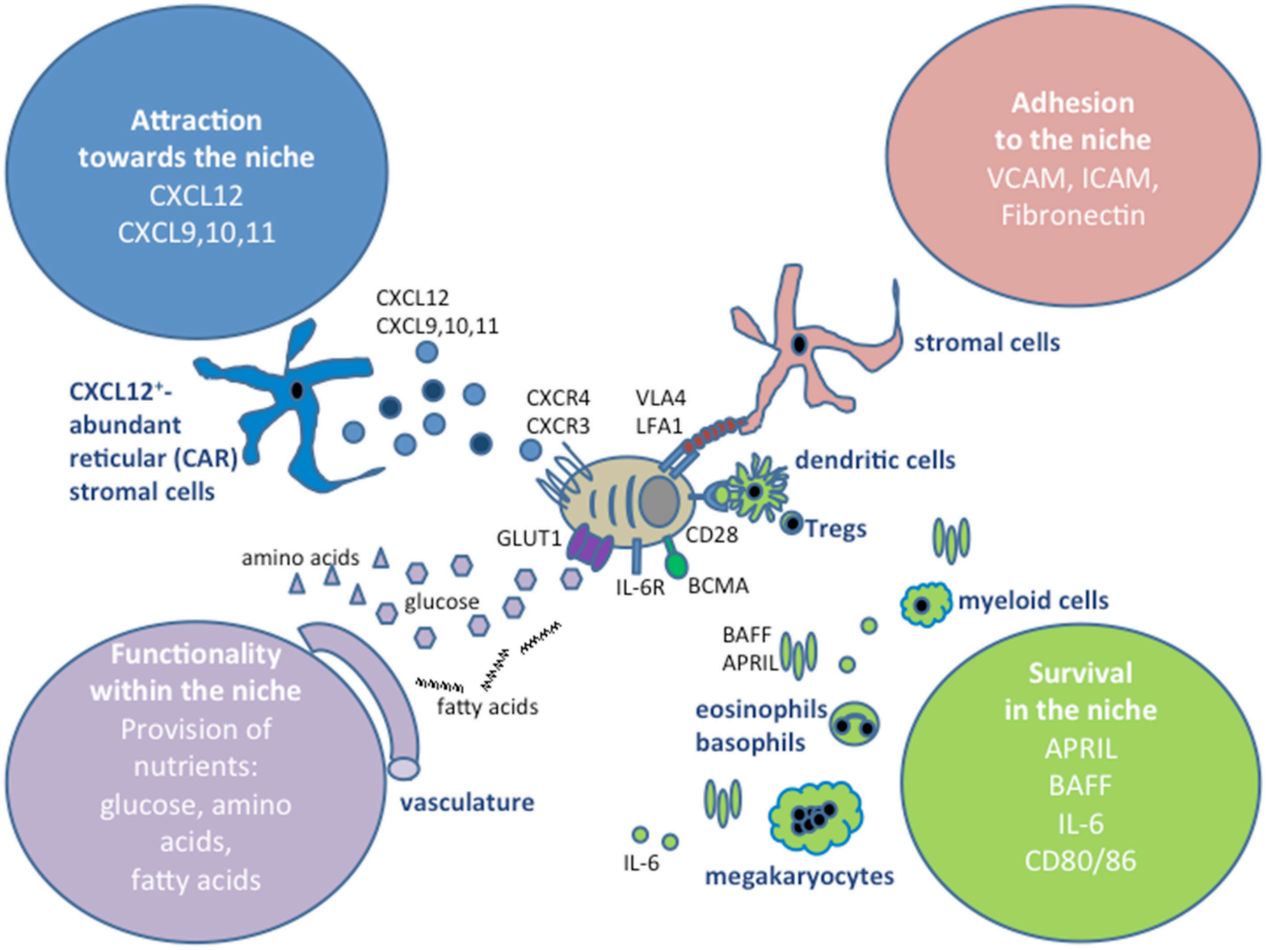
Extrinsic factors defining plasma cell niches. The extrinsic signals can be grouped into four categories, depending on whether they affect the migration of plasma cells into the niches, retain plasma cells at those sites, promote their survival or impact on their functionality, for example by providing nutrients. In this graph, the cell types that have been shown to contribute to the niches in the bone marrow are shown, however, the cells types involved in securing these four categories vary between the tissues where plasma cells are localized, as discussed in the text.

Due to its function in hematopoiesis, with blood cells constantly being generated and leaving the tissue, the bone marrow is a very dynamic tissue (55), with far more cells being generated and leaving than cells remaining resident and resting in this organ. On the order of a hundred million cells per gram of bone marrow are produced per day. Other hematopoietic cells which contribute to the niches may be only transient niche inhabitants, as has been shown for eosinophils (36), and the transient or migratory cells may not need to physically contact plasma cells. For example, APRIL can be produced in a soluble form by cells that are not in contact with plasma cells, and a local accumulation of APRIL in the niches could be achieved by its retention on the tissue via proteoglycans (56) or by directly binding to syndecan-1 (CD138) on the surface of plasma cells (57). Later in this review, we will address some interesting new findings about the dynamics of the bone marrow, and how this relates to its cellular and structural components. A summary of signals that have been described to promote plasma cell survival in the bone marrow is shown in Figure 1.

## Lifetime and Niches of Plasma Cells Generated in Intestinal Immune Responses

In contrast to their counterparts in the bone marrow, antibody-secreting cells in the intestine, the largest compartment of these cells in the body, were for a long time considered to be short-lived. Intestinal B cells migrate from the sites of their activation, Peyer's patches or mesenteric lymph nodes, through the blood to the lamina propria underlining the intestinal epithelium, where they secrete their antibodies, mostly IgA, at the frontline of defense. Their migration is guided by the chemokine receptors CCR9 and CCR10 (58). The high rate of proliferation among intestinal antibody-secreting cells led to the conclusion that these IgA+ cells have an average life span of less than a week in mice (59). However, studies in rats demonstrated the presence of a small, non-proliferative population of antibody-secreting cells (60). In mice, EdU pulse-chase experiments showed that long-lived IgA^+^ plasma cells are indeed generated in mucosal immune responses after oral immunization (61), although the short-lived plasma cells outnumber their long lived counterparts in the lamina propria, reflecting the continuous antigenic stimulation occurring at the mucosal interface. Plasma cells generated in intestinal immune responses also contribute to the long-lived plasma cell pool in the bone marrow.

Both intestinal and bone marrow long-lived plasma cells persist without antigenic stimulation (62, 63). The molecular structure of the survival niches in the gut is quite similar to the ones in the bone marrow, although the cells providing this structure are tissue-specific and thus quite different: the intestinal epithelium appears to be the main source of APRIL in mice (61) as well as humans (64). Eosinophils (45) and neutrophils (56) in the intestine also produce APRIL.

Human plasma cells in cultures of intestinal biopsies have been shown to survive without proliferation and secrete antibodies over several weeks, in a BCMA and IL-6 dependent manner (65). By using the analysis of cellular turnover in transplanted intestines and by calculating the age of individual cells using carbon-14 in DNA, the same lab showed that CD19^−^ plasma cells showed little to no turnover and persisted for decades, while CD19^+^ plasma cells were dynamically exchanged (66). Plasma cell populations differing in their CD19 expression have also been described in the bone marrow (67), with the CD19^−^ subset containing a static population providing long-term immunity, whereas the CD19^+^ subset appears to have more dynamic features of cells responding to a new antigenic challenge (68). The finding of heterogeneity among long-lived plasma cell populations is intriguing, and it will be interesting to investigate whether they differ in terms of requirements regarding their microenvironment, or whether they can occupy the same niche. Similarly, it will be important to investigate whether the recently described IL-10 producing subset of natural regulatory plasma cells (69) occupies a specific niche.

## Plasma Cells in Inflammatory Niches

Bone marrow and the intestine represent physiological locations for long lived plasma cells, however, plasma cells can also accumulate in chronically inflamed tissues. In autoimmune diseases like systemic lupus erythematosus (SLE), immune thrombocytopenia, autoimmune hemolytic anemia, and myasthenia gravis, plasma cells secreting autoantibodies are critical contributors to pathogenesis (70). Plasma cells have been shown to localize in inflamed kidneys of lupus-diseased NZB/W F1 mice (71) as well as in SLE patients (72, 73). The majority of the kidney plasma cells in the mouse model are long-lived (74), and they contribute to the local production of renal autoantibodies (75). Anti-dsDNA and –nucleosome autoantibodies have been shown to be present in glomerular deposits, a hallmark of nephritis (76, 77), although plasma cells secreting other specificities are also found in inflamed NZB/W F1 kidneys (71).

The long-lived autoreactive plasma cells pose a problem for therapy of these diseases, as they have been shown to be refractory to conventional immunosuppressive therapies (78) and downregulate B cell markers commonly used for therapy with B cell-depleting biologicals, such as CD20 (79). In fact, in immune thrombocytopenia patients treated with anti-CD20 (Rituximab), long-lived plasma cells are found to persist in the spleen (80), whereas they are absent in spleens of non-treated patients. Similar reports have also been observed in warm auto-immune hemolytic anemia patients (81). Plasma cell persistence in the B cell-depleted patients is accompanied by elevated splenic BAFF levels, which may indicate that the decreased local BAFF consumption (81, 82) permits *de novo* generation of niches for long-lived plasma cells in the spleen. Conversely, although the proteasome inhibitor bortezomib was shown to effectively deplete both short- and long-lived plasma cells in NZB/W F1 mice (83), as normal cellular turnover leads to continuous antigenic exposure to nucleosomes and other self-antigens, long-lived plasma cells are continuously generated in SLE-prone mice (84) and patients (85), and sustained depletion can only be achieved by combining bortezomib and B cell depletion (86). Together, this demonstrates that an effective therapy must take into account the interdependence of the various B cell differentiation stages and their requirements for cytokines.

Autoantibody-mediated diseases and the option to treat them using plasma cell depletion have gained increasing attention in the field of neurology in recent years. The central nervous system (CNS) represents a special segregated niche for plasma cells as well as for antibodies, as the blood-brain barrier separates the CNS tissue and cerebrospinal fluid from systemic blood flow in healthy individuals. Anti-N-methyl-D-aspartate receptor (NMDAR)-encephalitis is a recently described autoantibody-mediated immune disease. Patients present with heterogeneous symptoms, ranging from seizures and dyskinesia to frank psychosis, and antibodies against the NR1-subunit of the NMDAR have been shown to be pathogenic (87). Bortezomib reduced antibody titers and improved the clinical outcome in NMDAR patients resistant to other therapies (88). This suggests plasma cell targeting is an option also for the treatment of neuroinflammatory diseases such as neuromyelitis optica (NMO), which is clearly associated with autoantibodies specific for the water channel protein aquaporin 4 (AQP4), as well as with antibodies against myelin oligodendrocyte protein (MOG) in a subset of patients (89). Oligoclonal bands, indicative of intracerebral antibody production, have been regarded as hallmarks in the diagnosis of multiple sclerosis (MS) for decades (90), and B cell-depleting therapies are now utilized successfully to treat MS at least as effectively as conventional therapies [reviewed in (91)]. Axopathic and demyelinating antibodies are present in a subset of patients with MS, but there seems to be a high degree of mechanistic heterogeneity in this disease (92) and plasma cells may also have a role in ameliorating neuroinflammation by providing anti-inflammatory cytokines (93). In experimental autoimmune encephalomyelitis, a mouse model for MS, plasma cells accumulate in the CNS during peak disease and persist in the chronic state (94). Although the actual lifetime of plasma cells in CNS tissue from MS patients has not yet been determined, the fact that oligoclonal bands in the CSF recognize the same epitopes over time (90), together with the finding that CD138^+^ cells in brain tissue from MS patients are non-dividing (94), suggests that plasma cells persist in chronically inflamed human CNS. In EAE, the finding that tissue-specific, resident CNS cells such as astrocytes contribute to the formation of these niches supports the idea that various cell types in many different tissues can support the retention of plasma cells and transform into plasma cell niches, and that chronic inflammation can induce this process, even in tissues that are void of peripheral immune cells in healthy individuals.

Taken together, strategies for the future include a more selective targeting of plasma cells within the inflammatory niches, but this requires a more detailed knowledge of the cell types forming the niches in various organs under a range of healthy and pathologic conditions. For example, the niches for long-lived plasma cells in inflamed lupus kidneys are not well characterized. A recent publication reported the presence of APRIL-producing macrophages promoting plasma cell accumulation in renal lesions of patients with IgG4-related disease (95), but whether these macrophages are also abundant in kidneys from SLE patients remains to be investigated. The adhesion and survival signals required by plasma cells for persistence are provided by the niche, and are independent of the tissue of residence. As chronic inflammation can promote niche formation in widely varying tissues, the mechanisms by which the various cell types present in these tissue niches provide these signals and thereby affect plasma cell function may also have commonalities.

This review is focused on plasma cells, however, their malignant counterparts, multiple myeloma cells, were actually the first cells that could be efficiently targeted and eliminated, by therapeutically exploiting their high levels of protein production. using proteasome inhibitors. Bortezomib has been clinically used to treat patients with multiple myeloma for over a decade. In 2008, Voll et al. for the first time used Bortezomib to successfully target plasma cells in humoral autoimmunity, using a mouse model of SLE (83). Since then, it has been successfully used to treat human SLE (85, 96) and therapy-refractory anti-NMDA receptor encephalitis (88). More specific proteasome inhibitors such as carfilzomib and ixazomib have been developed, to reduce side effects. As plasma cells no longer express most of the surface molecules characteristic of the B cell lineage, they cannot be targeted by antibodies or cell-based therapies directed against B cells, for example CD20 or CD19 (79, 97). However, cell therapies targeting alternative antigens such as BCMA have demonstrated pre-clinical success and are in clinical trials (98, 99).

The bone marrow stromal microenvironment has been shown to support myeloma cell survival, immune evasion and expansion via contact-dependent mechanisms as well as the secretion of soluble mediators, by inducing the expression of transcription factors such as c-Myc, JunB, and c-Maf, hypo-methylation of histone H3 in promoter regions of anti-apoptotic genes such as *IGF-1* and *Bcl2*, and by modulating miRNA expression. Therefore, future therapeutic strategies should also include targeting the interaction between malignant plasma cells and their microenvironment [reviewed in (100)].

## Impact of the Microenvironment on Plasma Cell Metabolism and Function

Lymphocytes' activation states are tightly coupled to their metabolism. Resting cells have relatively low metabolic and synthetic requirements, and typically use oxidative metabolism (101, 102). Upon activation, lymphocytes increase their metabolic and synthetic capacity in preparation for proliferation. Plasma cells, due to their high biosynthetic demands, require an adequate supply of glucose and amino acids. Recent studies examining the metabolism of plasma cells have shown that although plasma cells take up large amounts of glucose, this is predominantly used for antibody glycosylation, not for metabolism (103). Both short- and long-lived plasma cells had similar basal oxidative respiration *in vitro*, but long-lived bone marrow plasma cells had a markedly higher oxidative respiratory capacity, as measured by oxygen consumption after addition of the ionophore FCCP (103). *In vivo*, disruption of the mitochondrial pyruvate transporter Mpc2 led to shortened lifespan and loss of long-lived plasma cells, and decreased antigen-specific antibody titers. Although bone marrow plasma cells used long-chain fatty acids for basal respiration, there were no evident differences between the mitochondrial size or potential in short- or long-lived plasma cells. However, despite their basal use of oxidative respiration, long-lived bone marrow plasma cells took up more glucose than short-lived splenic plasma cells, irrespective of proliferative status (103).

Although the bone marrow is heavily perfused, a significant fraction of the parenchyma is markedly hypoxic. Technical measurements of oxygen tension within tissue are difficult, as invasive measurements with a microelectrode invariably compromise the tissue integrity. Most of the data on tissue oxygen levels is therefore indirect, using expression of hypoxia-induced proteins, or binding of the exogenous hypoxia label pimonidazole, which covalently binds to cellular macromolecules at oxygen tension < 10 mmHg, and is detectable by immunostaining (104). An optical method using the phosphorescence lifetime of a platinum porphyrin-coumarin-343 nanoparticle was used to visualize the oxygen tension within calvarial bone marrow (105), which unexpectedly found that the endosteal region is less hypoxic than the perisinusoidal regions, due to small penetrating arterioles. This was somewhat controversial, as it had previously been reported that the endosteum was more hypoxic than the perisinusoidal regions, due to the relatively small diameter of the endosteal arteries (106, 107). This same report used the subset of perivascular Nestin^+^ cells that define the quiescent HSC niche to demonstrate that the pO2 was higher in the Nestin^+^ vessels near the bone surface than in the larger-diameter, Nestin^−^ vessels deeper in the bone marrow cavity. This suggests that HSC quiescence is not regulated by hypoxia *per se*, and raises the possibility that the relative lack of cell division in the Nestin^+^ niches may lead to a comparatively low usage and demand for O_2_, and thereby contribute to the relative hyperoxia of the Nestin^+^ niche.

It should be noted that these measurements were only conducted over a limited time frame, due to technical limitations of the cranial bone marrow imaging window; it was therefore not possible to image the same region over a prolonged interval to examine the degree to which the oxygen tension in individual regions varies over time.

**Figure 2:**
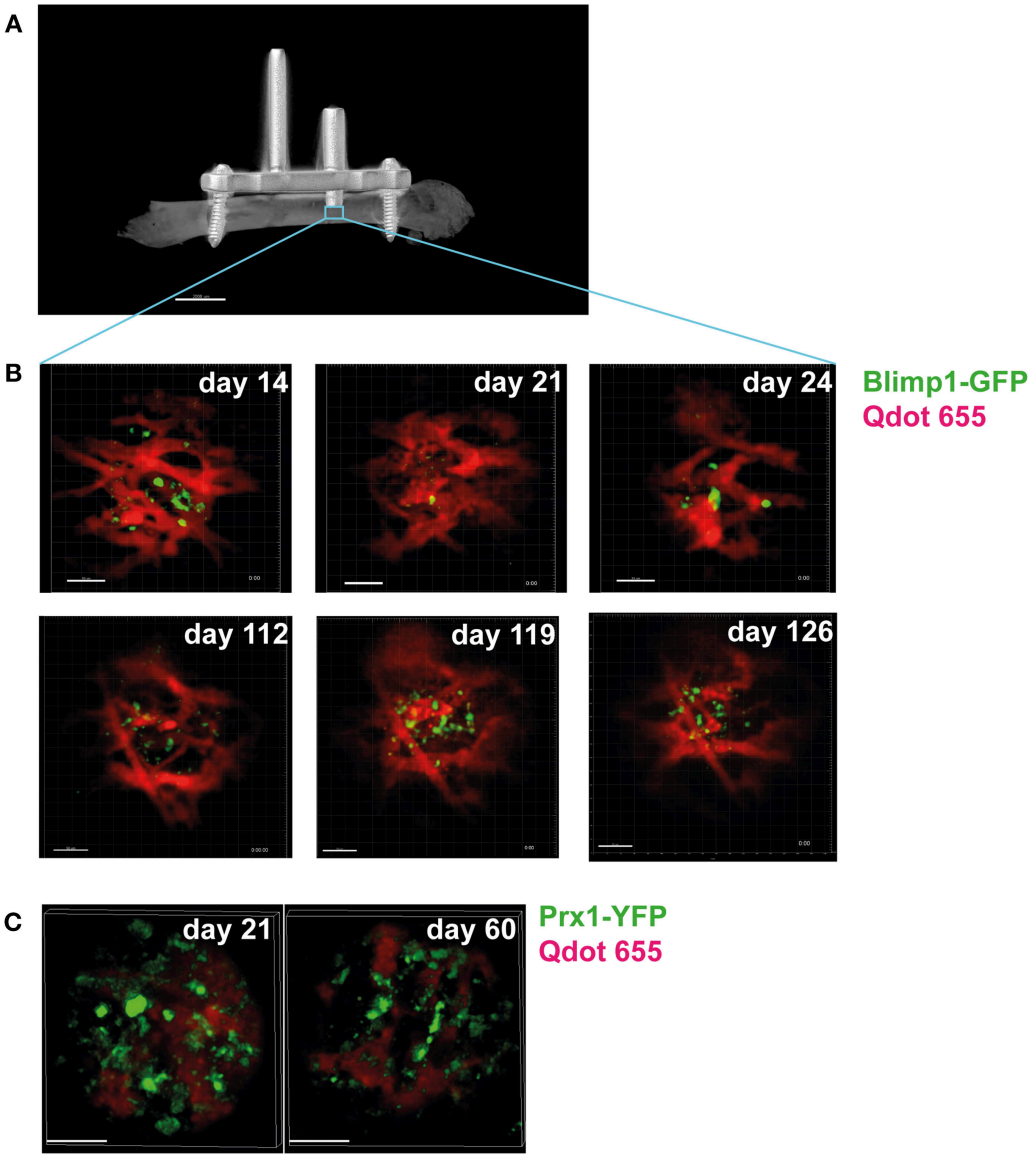
Longitudinal intravital imaging of plasma cells in bone marrow niches. **(A)** Micro-CT image of a mouse femur with an implanted device enabling longitudinal intravital microscopy of the bone marrow (LIMB). This device consists of a titanium-alloy based internal fixator used to fix an imaging port (arrow), which holds a gradient-refractory index (GRIN) lens onto the femur of live mice (55). The lens can be installed to reach deep into the bone marrow, thereby allowing imaging of tissue areas previously inaccessible by intravital microscopy (blue square), such as plasma cell niches, and allows imaging of the same area over months. **(B)** Longitudinal imaging of plasma cell niches reveals dramatic changes in the vascular structure (red) surrounding the plasma cells (green) over time. Time points (days) after implantation of the device are indicated. Scale bar 50 μm. **(C)** Changes are also present in the stromal compartment, as revealed by imaging of reporter mice expressing yellow fluorescent protein in bone marrow stromal cells [Prx-Cre (108) x R26R-eYFP (109)]. Stromal cells are shown in green, vessels in red. Scale bar 50 μm.

Recently, a method developed by our group (55) that permits longitudinal imaging of bone marrow over the time course of several months has demonstrated that the structure of the blood vessels in the bone marrow dynamically changes, on a time scale of days (small vessels) to weeks (larger vessels). This is also evident in vessels surrounding bone marrow plasma cells (Figure 2). This strongly suggests that the variability in the provision of nutrients and O_2_ does not only depend on spatial conditions, but also changes over time. It is currently unclear how long-lived plasma cells deal with these changes, since they have a high biosynthetic demand, given that they can secrete >1,000 antibody molecules per second (110). To permit this massive amount of protein synthesis and secretion, plasma cells dramatically up-regulate the unfolded protein response (111, 112). Autophagy is one component of this, which is required for the survival of long-lived plasma cells in the bone marrow (113). Cre-mediated deletion of the autophagy factor Atg5t in B cells led to increased ER size and ER stress signaling, which led to higher BLIMP-1 expression and increased antibody secretion (114). However, this higher-level antibody synthesis caused a pronounced decrease in viability, leading to lower antibody responses and markedly lower numbers of long-lived bone marrow plasma cells, with the plasma cells in the bone marrow all having escaped Atg5 deletion (114).

This up-regulation of the unfolded protein response is intrinsic to the plasma cell fate, and is controlled by the master plasma cell transcription factor Blimp1 (115). Interestingly, high-level antibody secretion is not necessary for plasma cell survival, as deletion of the unfolded protein response-related transcription factor Xbp1 specifically in B-lineage cells led to markedly reduced antibody synthesis, but no appreciable differences in plasma cell proliferation, differentiation, or cell number in both resting and immunized mice (116). This is consistent with the observation that the rate of antibody secretion is not uniform across all plasma cells (117). It may suggest that plasma cell antibody synthesis should not be expected to be constant over time, but to vary widely in all plasma cells, and to be influenced by the perfusion and oxygenation status of the plasma cell niche. The finding that inhibition of the cellular nutrient sensor mammalian target of rapamycin complex 1 (mTORC1) blocks antibody synthesis without affecting long-lived plasma cell survival is very interesting in this context (118). Of note, mTOR-inhibition not only prevents the development of lupus nephritis (119), it also effectively attenuates established disease in NZB/W F1 mice (120).

## Toward Understanding the Link Between Plasma Cell Metabolism and Function *in vivo*: New Tools on the Horizon

Taken together, there is a strong demand for methodologies that allow monitoring of plasma cell metabolism over time *in vivo*. In general, metabolically active cells have less free NAD(P)H and more enzyme-bound NAD(P)H. It has been known since the 1960s that the fluorescence of the endogenous cofactor NAD(P)H can be used to measure the metabolic activity of cells (121). The fluorescence lifetime of NAD(P)H gives information about the relative amounts of free and protein-bound NAD(P)H, and reflects the enzymes to which NAD(P)H is bound (122, 123). Actively proliferating cells have been shown to have more free NAD(P)H, in addition to a longer fluorescence lifetime (124, 125). However, malignant cell lines have shorter NAD(P)H fluorescence lifetimes than relatively benign, more slowly-proliferating cell lines. As the fluorescence lifetime for free NAD(P)H is markedly shorter than for enzyme-bound NAD(P)H, this indicates that there is not a simple 1:1 correspondence between cell proliferation and shorter NAD(P)H half-lives. Instead, it will depend on the specific NAD(P)H-binding enzymes in the cells of interest: on their NAD(P)H fluorescence lifetimes, and the extent to which they are bound.

**Figure 3:**
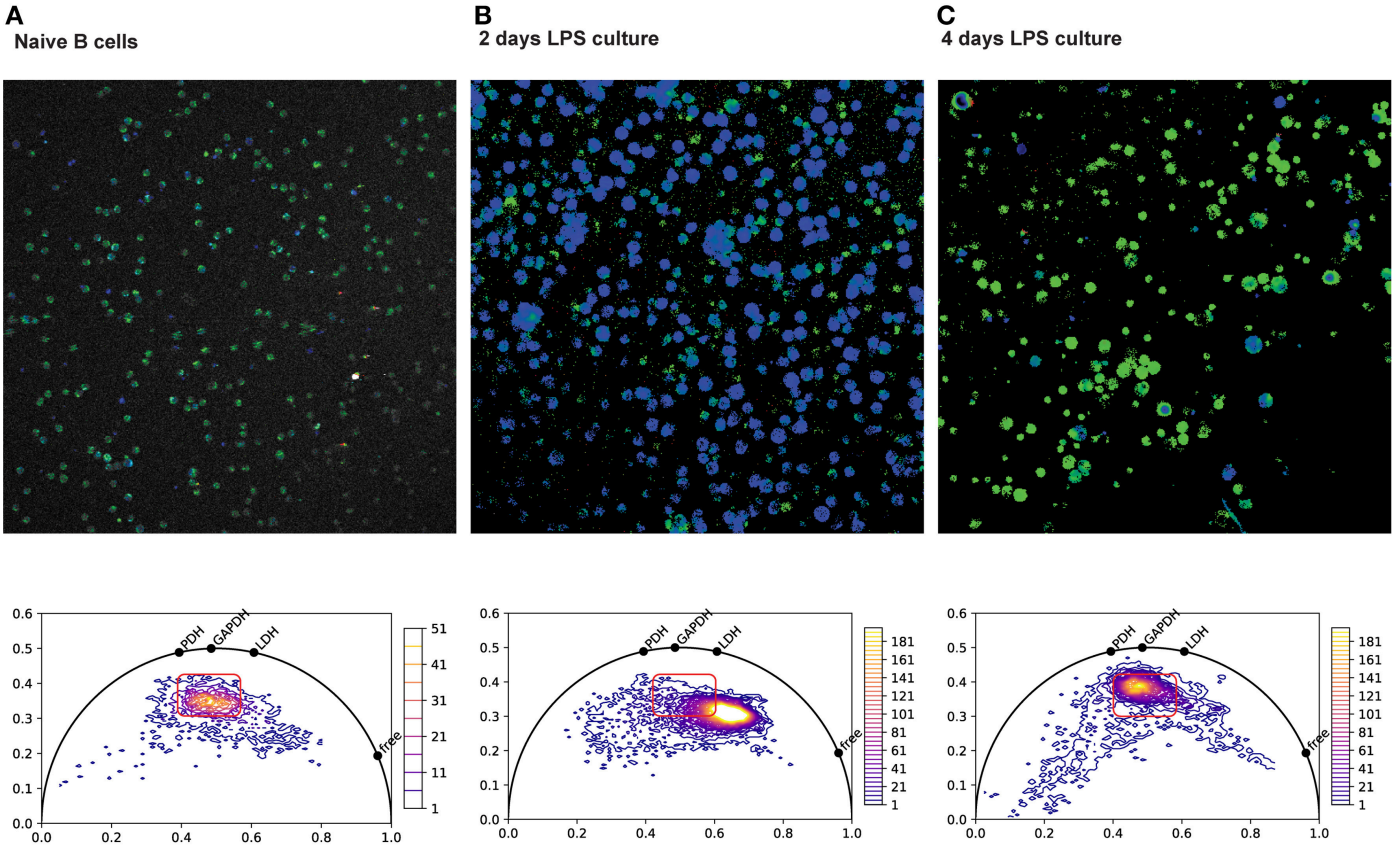
NAD(P)H fluorescence lifetime imaging (FLIM) of splenic plasma cells. Top row: Color-coded NAD(P)H fluorescence lifetime images of splenic B cells isolated by negative immuno-magnetic selection, imaged directly (**A**, naive), or after two **(B)** or four **(C)** days of culture in LPS. Bottom row: Corresponding phasor plots of NAD(P)H fluorescence lifetimes from the images in the top row. Phasor plots are generated from the discrete Fourier transform of the fluorescence decay at each pixel in the image. Points on the semicircle correspond to mono-exponential decay, and points within the semicircle correspond to the intensity-weighted vector sum in of their components. Shorter decay times are along the right of the semicircle, and longer decay times are to the left (132). Hence, pure free NAD(P)H with a fluorescence lifetime of ~400 ps appears further to the right on the phasor semicircle than NADH bound to lactate dehydrogenase (LDH) at 1,600 ps, NADH bound to gyceraldehyde-3-phosphate-dehydrogenase (GAPDH) at 2,050 ps and NADH bound to pyruvate dehydrogenase (PDH) at 2,470 ps. After 2 days of LPS culture, the rapidly proliferating cells show a marked shift toward shorter NAD(P)H lifetimes, consistent with more free NAD(P)H and a more glycolytic metabolism dominated by LDH.

We and others have recently developed methods to “fingerprint” specific NAD(P)H –binding enzymes, using the lifetime of NAD(P)H fluorescence (126–131), and are applying these methods to visualize the metabolic status of single cells in tissue. Our data demonstrates that B cell blasts have a markedly shorter NAD(P)H half-life than do naive splenic B cells (Figure 3) (133). As cell proliferation slows down, the NAD(P)H lifetime lengthens again, to values similar to those of naive B cells. This is consistent with increased levels of free NAD(P)H in rapidly proliferating cells, as previously reported (124, 125), in addition to a shift from oxidative to glycolytic metabolism. Bone marrow plasma cells, sorted and analyzed *in vitro* (data no shown), had a fluorescence lifetime similar to that of naive B cells, consistent with a largely oxidative metabolism. Although plasma cells take up large amounts of glucose, as it is used for antibody glycosylation (103), it has no bearing on the cellular metabolic status.

Despite being a promising approach for deciphering the metabolic activity of cells *in vivo*, in tissue context, NAD(P)H fluorescence lifetime imaging (FLIM) has been used only in the last few years for this purpose, with most of the studies investigating single time points (127–129, 134). As of this writing, there have been no NAD(P)H-FLIM studies concerned with the metabolism of long-lived plasma cells in the bone marrow. The lack of *in vivo* NAD(P)H-FLIM studies is due to technical limitations in acquiring and analyzing images of fluorescence lifetimes that have only recently been overcome. For example, frequency-domain FLIM technologies are prone to numerical artifacts when analyzing complex exponential fluorescence decays (135). Further, they are based on field-detection using cameras, which makes their use in highly scattering media such as tissue very difficult. Only a few years ago, time-domain FLIM technologies, of which time-correlated single-photon counting is the best suited for deep-tissue *in vivo* studies (126), relied on analyzing less than 5% of the emitted photons. This necessarily required extremely high photon counts, incompatible with deep-tissue or dynamic imaging. First the parallelization of detection and photon counting and the use of hybrid photomultiplier detection in FLIM led very recently to the successful acquisition of time-lapse FLIM movies (126, 130). These time-lapse FLIM experiments make us confident that NAD(P)H-FLIM combined with longitudinal bone-marrow imaging will open new insights into the enzymatic and metabolic lifestyle of long-lived plasma cells. There are significant technical challenges in measuring NAD(P)H fluorescence lifetimes *in vivo*, in particular the relatively low intrinsic fluorescence of NAD(P)H, and the high density of cells. However, despite these challenges, *in vivo* NAD(P)H FLIM remains the most promising method of imaging the metabolic status of single cells, and provides an unparalleled view into the metabolic and functional dynamics of cells in their native tissue context-especially the plasma cell niche.

## Concluding Remarks

Recent progress in the fields of immunometabolism and single-cell analysis and has led to an improved understanding of plasma cell biology. Due to the importance of the microenvironment in promoting plasma cell function and survival, functional analyses of plasma cells in the tissue context will be key in order to fully understand their lifestyle. This will be critical in developing better ways to target pathologic plasma cells in autoimmunity, malignant plasma cells in multiple myeloma, and also to enhance antibody secretion to improve the efficacy of vaccination.

## Author Contributions

RL generated NAD(P)H-FLIM data. RN and AH generated longitudinal intravital imaging data of the plasma cell niches. All authors reviewed literature and contributed to writing of the manuscript.

### Conflict of Interest Statement

The authors declare that the research was conducted in the absence of any commercial or financial relationships that could be construed as a potential conflict of interest.

## Abbreviations

Abs: Antibodies
APRIL: A proliferation-inducing ligand
ASC: Antibody-secreting cell
BAFF: B-cell activating factor
BCMA: B cell membrane antigen
CNS: Central nervous system
CSF: Cerebrospinal fluid
CXCL: Chemokine X ligand
CXCR: Chemokine X receptor
EAE: Experimenal autoimmune encephalitis
ER: Endoplasmic reticulum
FCCP: Carbonyl cyanide-p-trifluoromethoxyphenylhydrazone
FLIM: Fluorescence lifetime imaging microscopy
GAPDH: gyceraldehyde-3-phosphate-dehydrogenase
GC: germinal center
GRIN: Gradient refractive index
GTI: Germinal center-T cell zone interface
HSC: hematopoietic stem cell
HSPC: hematopoietic stem and progenitor cell
LDH: lactate dehydrogenase
LIMB: Longitudinal imaging of the bone marrow
MC: medullary cords
MOG: myelin oligodendrocyte glycoprotein
MS: multiple sclerosis
mTOR: mammalian target of rapamycin
NADH: Nicotinamide adenine dinucleotide
NADPH: Nicotinamide adenine dinucleotide phosphate
NAD(P)H: either NADH or NADPH
NMDAR: N-methyl-d-aspartate receptor
NZB/W F1, New Zealand black × New Zealand white: F1 generation
PC: Plasma cell
PDH: Pyruvate dehydrogenase
RNA: Ribonucleic acid
RP: red pulp
SLE: systemic lupus erythematosus
SLO: Secondary lymphoid organ
VCAM: Vascular cell adhesion molecule.
